# VisR Ultrasound With Non-Normal ARF-AoS Incidence Interrogates Both Shear and Young’s Elastic Moduli in Transversely Isotropic Materials

**DOI:** 10.1109/ojuffc.2024.3520516

**Published:** 2024-12-19

**Authors:** SABIQ MUHTADI, CATERINA M. GALLIPPI

**Affiliations:** Joint Department of Biomedical Engineering, The University of North Carolina (UNC) at Chapel Hill, Chapel Hill, NC 27599 USA North Carolina State University (NCSU), Raleigh, NC 27695 USA

**Keywords:** Ultrasound elastography, acoustic radiation force impulse (ARFI), viscoelastic response (VisR) ultrasound, transversely isotropic (TI) materials, shear elastic modulus, Young’s modulus

## Abstract

This study evaluates the potential for interrogating the Young’s elastic moduli in anisotropic media, including tissue, using Viscoelastic Response (VisR) ultrasound. VisR is an on-axis acoustic radiation force (ARF)-based elasticity imaging method that has been demonstrated previously for assessing the shear elastic moduli of transversely isotropic (TI) materials when the applied ARF excitation was incident normal to the axis of symmetry (AoS). It is hypothesized that by applying a range of non-normal ARF excitations and monitoring the percent change in VisR-derived relative elasticity (RE) versus ARF-AoS incidence angle, both the shear and the Young’s elastic moduli may be interrogated. The hypothesis was tested using *in silico* experiments, which showed that while RE measured at normal ARF-AoS incidence was related to only longitudinal shear modulus $\left(\mu_{L}\right)$ alone, the percent change in RE over ARF-AoS incidence angle (defined as ΔRE) exhibited a strong linear correlation with the ratio of longitudinal shear-to-Young’s moduli $\left(\mu_{L}/E_{L}\right)$, with correlation coefficients of 0.97–0.99. Additionally, the linear regression slopes of ΔRE versus ARF-AoS incidence angle statistically discriminated TI materials with $\mu_{L}/E_{L}$ ratios that were as little as 7% different. These findings were validated *ex vivo* in chicken *pectoralis major* and bovine *longissimus dorsi* muscles, where the rate of change in of ΔRE versus ARF-AoS incidence angle distinguished the two muscles with statistical significance. The results obtained in this study suggest that the rate of change of ΔRE with ARF-AoS incidence angle may serve as a novel, semi-quantitative biomarker for characterizing anisotropic tissues such as kidney, skeletal muscle, and breast.

## I. INTRODUCTION

Ultrasound Elastography (UE) techniques provide a non-invasive means to assess tissue stiffness [1], which is valuable for characterizing soft tissues that have undergone elasticity changes due to pathological or physiological processes [2]. Several of these techniques utilize acoustic radiation force (ARF) generated by focused ultrasound beams to induce localized mechanical perturbations within tissues, which are subsequently leveraged to evaluate underlying mechanical properties [3]. However, in the majority of such applications, the evaluated soft tissues are assumed to be locally homogenous and isotropic, such that their inherent mechanical properties do not vary with direction [4]. While this assumption may hold for some organs such as liver [5], it is important to note that most biological tissues including skeletal muscle, kidney, and breast are mechanically anisotropic [6], [7], [8]. In the simplest form, such mechanical anisotropy can be described as transversely isotropic (TI) [9], such that the shear and Young’s elastic moduli vary along versus across a material axis of symmetry (AoS).

**FIGURE 1.**
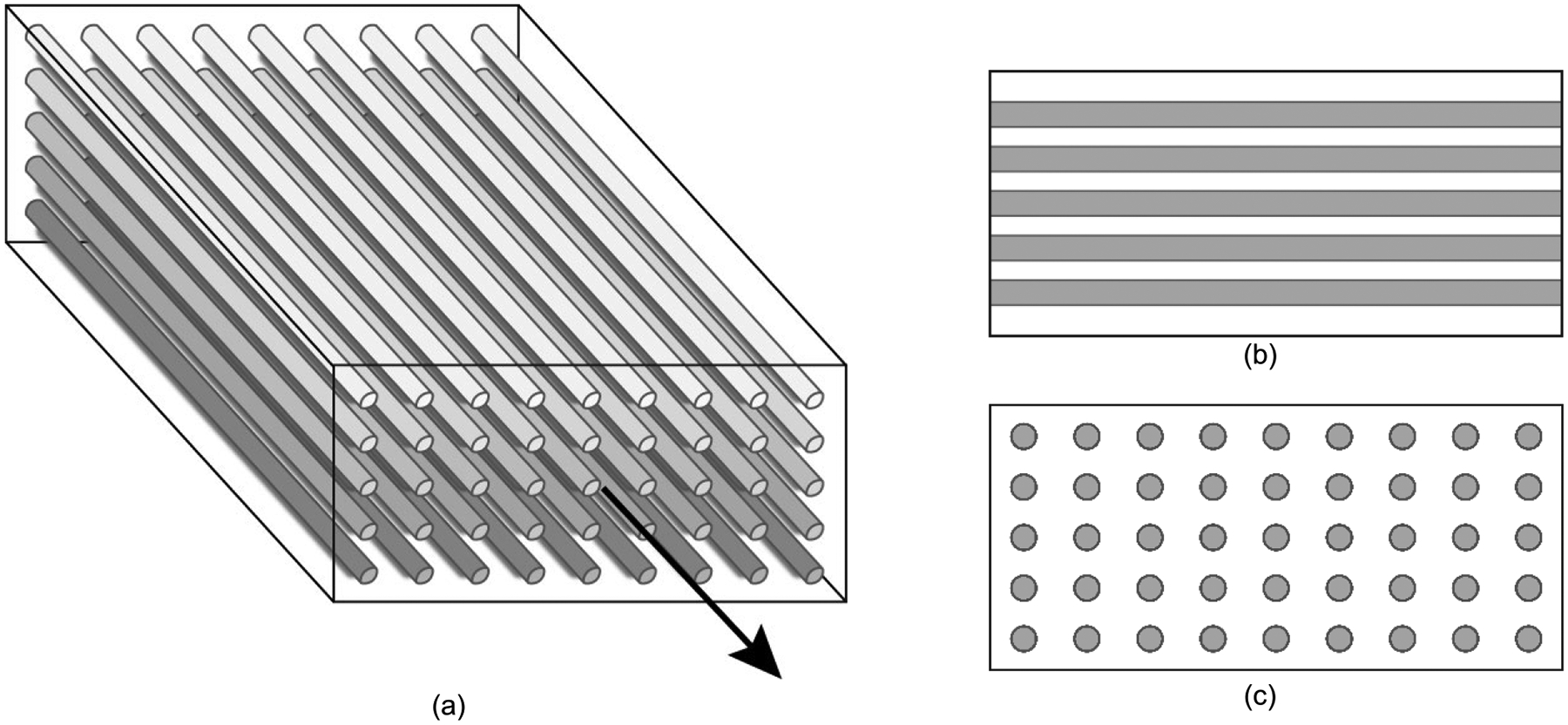
(a) Three-dimensional representation of a transversely isotropic (TI) material, with an axis of symmetry (AoS) (arrow), (b) plane of symmetry, and (c) plane of isotropy.

A representative TI material is illustrated in Fig. 1. The plane of symmetry and the plane of isotropy are oriented perpendicular to the AoS. While the mechanical properties of TI materials may be described in terms of Young’s and shear moduli, their interrelationship is more complex than in isotropic materials. The constitutive equation for a linear, elastic material can be written in terms of a generalized Hooke’s law between a stress tensor, σ, and a strain tensor, ε, as $\sigma_{ij}=c_{\text{ijkl}}\varepsilon_{kl}$, where $c_{\text{ijkl}}$ is a fourth-order stiffness tensor that characterizes the material. Due to rotational and reflective symmetries in a TI material, the stiffness tensor reduces from 81 elastic constants to five independent quantities [10], [11]. These five independent quantities can further be expressed in terms of six engineering constants and one constraint: two Young’s moduli oriented along and across the AoS in longitudinal (L) and transverse (T) directions ($E_{L}$ and $E_{T}$, respectively); two shear moduli, ($\mu_{L}$ and $\mu_{T}$); two Poisson’s ratios in the plane of isotropy and plane of symmetry ($v_{TT}$ and $v_{LT}$, respectively); and the constraint,

(1) $$\mu_{T}=\frac{E_{T}}{2\left(1+v_{TT}\right)}$$

Furthermore, if the material is incompressible, the Poisson’s ratios, $v_{TT}$ and $v_{LT}$, have specific values as follows [9], [10]:

(2) $$v_{TT}=1-\frac{E_{T}}{2E_{L}}$$

(3) $$v_{LT}=\frac{1}{2}$$

Due to these constraints, an incompressible TI (ITI) material can be described in terms of three material constants: $\mu_{T},\mu_{L}$ and $E_{T}/E_{L}$.

TI material properties have been evaluated using Viscoelastic Response (VisR) ultrasound, an on-axis, ARF-based, elastography technique [12], [13]. VisR ultrasound is performed using two successive, spatially co-localized ARF excitations, which generate micrometer-scale displacements to approximate a creep response in the ARF region of excitation (ROE) [12], [13]. The induced displacements are tracked ultrasonically and fit to a three-parameter mass-spring-damper (MSD) model, where one parameter accounts for elasticity, one for viscosity, and the last an inertial component, as described by the following second-order differential equation [14]:

(4) $$m\frac{d^{2}}{dt^{2}}z(t)+\eta \frac{d}{dt}z(t)+\mu z(t)=F(t)$$

where m[Kg] is mass, z(t)[m] is the axial displacement, $\eta \left[{\text{Nm}}^{-1}\;s\right]$ is the viscosity, $\mu \left[{\text{Nm}}^{-1}\right]$ is the shear modulus, and t[s] is the time. F(t) is the ARF excitation that is described in time as a rectangular function of force magnitude A and duration $t_{ARF}$ as follows:

(5) $$F(t)=A\left(H\left(t-t_{\text{ARF}}-t_{s}\right)-H\left(t-2t_{\text{ARF}}-t_{s}\right)\right)+A\left(H(t)-H\left(t-t_{\text{ARF}}\right)\right)$$

where H is the Heaviside function, and $t_{s}[s]$ is the time separating the ARF pushes. Substituting (5) into (4) results in:

(6) $$\frac{d^{2}}{dt^{2}}z(t)+\omega_{n}^{2}\tau \frac{d}{dt}z(t)+\omega_{n}^{2}z(t)\;=S\omega_{n}^{2}\left(H(t)-H\left(t-t_{ARF}\right)\right.\left.\;+H\left(t-2t_{ARF}-t_{s}\right)-H\left(t-2t_{ARF}-t_{s}\right)\right)$$

where $\omega_{n}\left[{\;s}^{-1}\right]$ is the natural frequency, τ[s] is the relaxation time constant, and S[m] is the static sensitivity of the system. The values of $\omega_{n},\;\tau$ and S are defined as:

(7) $$\omega_{n}=\sqrt{\frac{\mu }{m}};\tau =\frac{\eta }{\mu };S=\frac{A}{\mu }$$

Equation (6) is solved for the displacement z(t), and then unconstrained nonlinear optimization is used to fit the ultrasonically tracked displacements at each spatial location to the solution of (6) to estimate $\omega_{n},\;\tau$, and S. Next, relative elasticity $\left(\text{RE}\left[m^{-1}\right]\right)$ and relative viscosity $\left(\text{RV}\left[m^{-1}s\right]\right)$ are derived as:

(8) $$RE=\frac{1}{S}=\frac{\mu }{A}$$

(9) $$RV=\frac{\tau }{S}=\frac{\eta }{A}$$

Both RE and RV are considered relative to the applied ARF amplitude A, which is unknown but assumed to be constant over the imaging field-of-view (FOV).

Previous research has demonstrated that VisR RE measurements in TI materials vary as the lateral-elevational long-axis of the ARF point-spread-function (PSF) is rotated from being aligned across (longitudinal orientation) to along (transverse orientation) the AoS, and that the degree of this variation is proportional to the ratio of the underlying shear elastic anisotropy $\left(\mu_{L}/\mu_{T}\right)$. This effect has been demonstrated *in silico* [15], [16], as well as *in vivo*, with the ratio of VisR RE taken along versus across tissue AoS differentiating between patients with versus without Duchenne muscular dystrophy [17] and patients with benign versus malignant breast masses [18], [19]. However, these prior studies employed ARF excitations that had a 90° incidence angle with respect to the underlying tissue AoS, resulting in observed displacements that predominantly reflected shear elastic moduli [15], [16], [20]. Conversely, a different study found *via* finite element method (FEM) simulations and continuous loading experiments that when the incident angle of an applied force varied between 0° and 90° relative to the AoS of glass fiber reinforced polymer (GFRP)— a TI material — the resulting stiffness measurements reflected a combination of Young’s and shear elastic moduli [21]. Given these findings, it is hypothesized that VisR elasticity measurements taken in TI soft tissues with non-normal ARF-AoS incidence angles will reflect a combination of both Young’s and shear and elastic moduli ($E_{L}$ and $\mu_{L}$). This hypothesis is herein evaluated *in silico* using FEM simulations mimicking VisR acquisitions in TI media, and the method is translated to physical realization in excised bovine *longissimus dorsi* and chicken *pectoralis major* muscles.

## II. METHODS

### A. IN SILICO VisR SIMULATION FRAMEWORK

The *in silico* simulation framework was adapted from the methods developed by Palmeri et al. [22] and later implemented by Hossain et al. [15], [16]. The LS-DYNA3D (Ansys Inc., Canonsburg, PA, USA) FEM solver was used to predict TI material responses to VisR ARF impulses. Firstly, the acoustic intensity field for an ARF push characterized by the parameters provided in Table 1 was calculated using the Field II ultrasound simulation package [23]. The calculated intensity values were scaled to a peak intensity of 5000 W/cm^2^, and the radiation force magnitude was derived according to the following expression [24]:

(10) $$\vec{F}=\frac{2\alpha \vec{I}}{c}$$

where α is the absorption coefficient of the medium (assigned as 0.5 dB/cm/MHz for soft tissue), c is the speed of sound in the medium (assumed to be 1540 m/s as in soft tissue), and $\vec{I}$ is the temporal average beam intensity over a volume. As described in (10), point load forces were calculated by spatially sampling the volumetric radiation force $\vec{F}$.

Next, TI materials were defined using the LS-DYNA3D “MAT_ORTHOTROPIC_ELASTIC” material model. Twelve different TI materials were analyzed in this study. Their mechanical properties were chosen to coincide with those published for TI soft tissues such as skeletal muscle, kidney, and breast: $E_{T}=11.74-24.96\;\text{kPa},\;E_{L}=35.23-82.78\;\text{kPa},\;\mu_{T}=3.20-6.80\;\text{kPa}$, and $\mu_{L}=4.80-40.80\;\text{kPa}$ [25], [26], [27], [28], [29], [30], [31], [32]. Each material was defined for a finite element (FE) mesh that spanned 7 to 42 mm axially, −8 to 8 mm laterally, and −8 to 8 mm elevationally, with each element having dimensions of 0.2 × 0.2 × 0.2 mm^3^ [16]. Furthermore, for each TI material, a 6-element thick perfectly matched layer (PML) was implemented around the mesh using the LS-DYNA3D “MAT_PML_ELASTIC” material model. The purpose of the PML was to simulate an infinite medium and remove spurious wave reflections from the boundaries of the mesh [15], [16].

The previously calculated point loads were then applied to each material mesh twice to simulate the two VisR ARF pushes. The point loads’ magnitude was kept constant for 70 μs, and the ARF pushes were separated by 0.4 ms. Each material was assessed in two different orientations: (i) with the long axis of the lateral-elevational ARF PSF aligned across the material AoS (i.e., interrogating the plane of symmetry in longitudinal orientation), and (ii) with the long axis of the lateral-elevational ARF PSF aligned along the material AoS (i.e., interrogating the plane of isotropy in transverse orientation).

**FIGURE 2.**
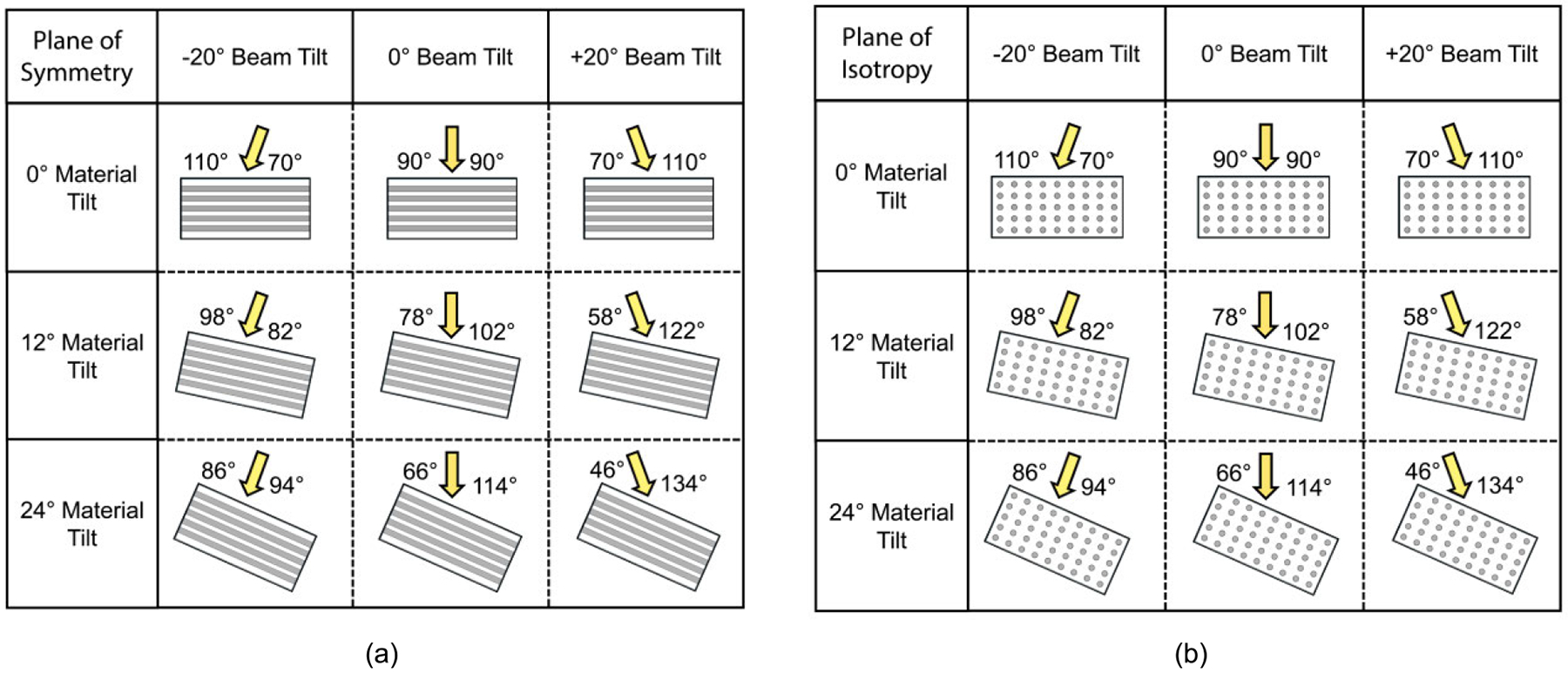
(a) The nine ARF-AoS incidence angles shown in the plane of symmetry, achieved using a combination of ARF beam and material tilting. (b) The same combinations of ARF-AoS incidence angles shown in the plane of isotropy.

In each orientation, variations in the ARF-AoS incidence angle were achieved by tilting the simulated ARF pushes to −20°, 0°, and 20° and by tilting the material by 0°, 12°, and 24°, for a total of nine incidence angles per orientation, as shown in Fig. 2(a) for the plane of symmetry and Fig. 2(b) for the plane of isotropy. The ±20° beam tilt was chosen to represent the practical maximum achievable using electronic steering of a linear-array transducer, while the material tilt further extended the ARF-AoS incidence angle range beyond that achievable by electronic steering alone. Note that, although it is expected that VisR RE will vary with ARF-AoS incidence angle in only the plane of symmetry, experiments in the plane of isotropy were included to confirm the expectation. Simulations were allowed to run for a total of 4.35 ms, and displacement data were sampled every 0.1 ms between the two ARF impulses and following the second ARF impulse to represent a conventional ultrasound imaging ensemble with a pulse repetition frequency (PRF) of 10 kHz [15], [16].

To simulate ultrasonic tracking, 3D scatterer phantoms were defined in Field II to span the volume of the FEM mesh. For each simulated TI material with a specific orientation and ARF-AoS incidence angle, ten unique scatterer realizations were generated (for a total of 180 unique iterations per TI material: 2 orientations × 9 ARF-AoS incidence angles × 10 unique scatterer realizations). The LS-DYNA3D derived displacements were used to linearly interpolate the scatterer positions for every time step in the VisR simulation ensemble using MATLAB (Mathworks Inc., Natick, MA, USA). After generating the scatterer position matrices for each time step, the corresponding RF lines were simulated using Field II. White Gaussian noise was added to each RF line using the *awgn* function in MATLAB to simulate a system SNR of 40 dB, which is typical of commercially available clinical ultrasound imaging systems.

Motion tracking was performed on the RF lines using 1D axial normalized cross-correlation (NCC) with parameters listed in Table 1 and using procedures described in [33]. A dataset describing axial displacements versus time was generated for each independent speckle realization, and the temporal displacement profiles were then fit to the MSD model using a custom C++ implementation of Nelder-Mead non-linear least squares minimization [12]. VisR RE values for each speckle realization were then derived according to (8), and RE measurements were analyzed in terms of mean and standard deviation of values from the same region-of-interest (ROI) (a 2 mm region about the focal depth) across the 10 speckle realizations for each unique TI material with specific orientation and ARF-AoS incidence angle.

### B. EX VIVO DATA ACQUISITION

**FIGURE 3.**
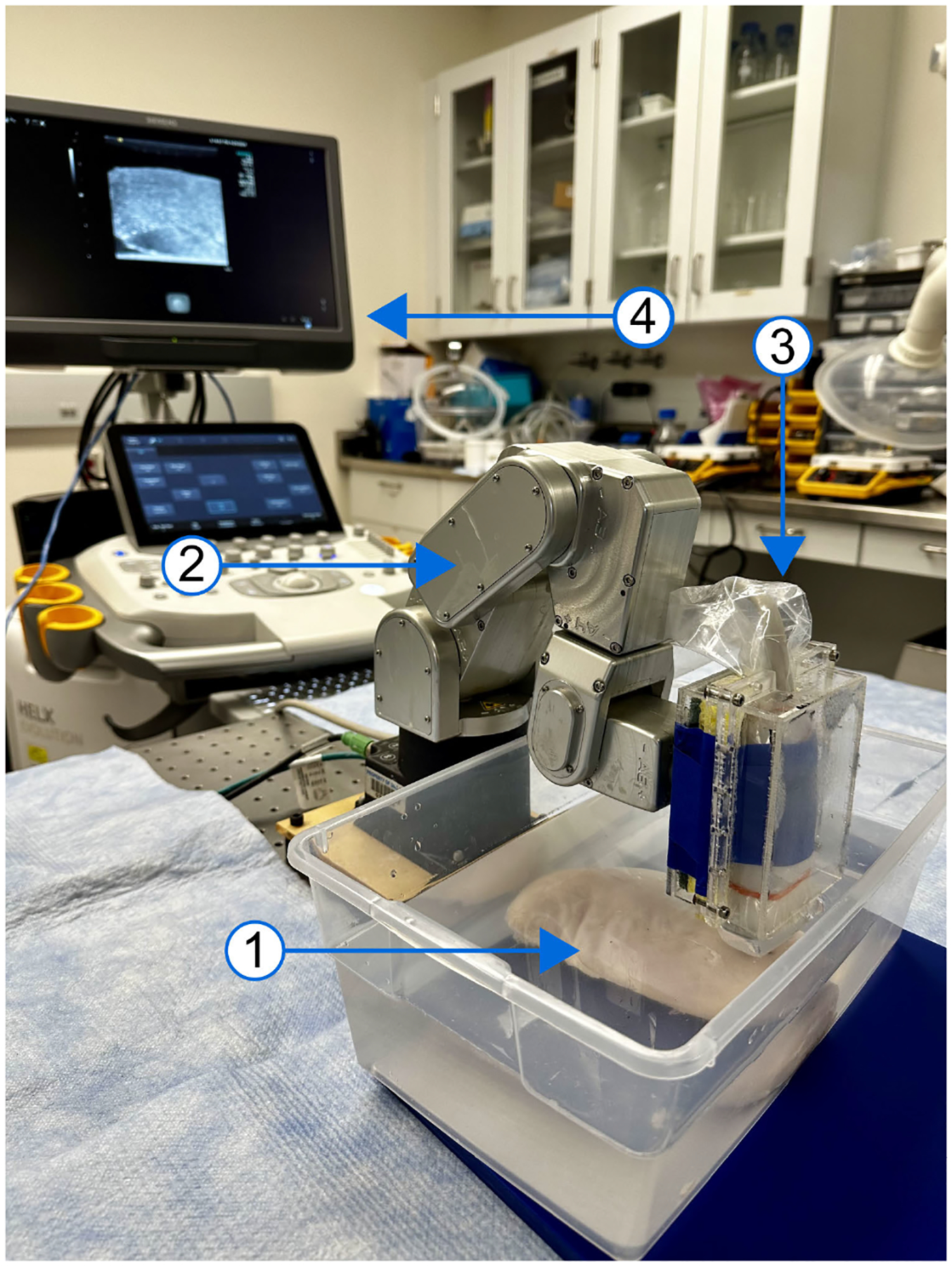
Experimental setup for acquiring *ex vivo* data from bovine *longissimus dorsi* and chicken *pectoralis major* samples labelled as follows: (1) *ex vivo* sample (chicken sample demonstrated), (2) 6-DoF serial robotic arm (Meca500, Mecademic, Montreal, Quebec, Canada), (3) 9-L4 transducer (Siemens Healthineers, Ultrasound Division, Issaquah, WA, USA) attached to robotic arm with custom transducer holder, and (4) S3000 Helix imaging system (Siemens Healthineers, Ultrasound Division, Issaquah, WA, USA). Data were acquired at normal (90°) ARF-AoS incidence followed by 10°, 20°, 30°, and 40° tilting of the transducer about a fixed center axis using the robotic arm.

To physically translate *in silico* results, *ex vivo* experiments were performed on bovine *longissimus dorsi* and chicken *pectoralis major* samples purchased from a local butcher shop. These muscles were chosen due to their structure and visible alignment of muscle fibers. Samples were kept at room temperature for 2–3 hours before being placed in a room-temperature water bath for imaging on a motion isolation table (Newport, Irvine, California, USA). For each sample, fiber orientation was determined by visual inspection of the surface and by B-mode imaging. Samples were then positioned to acquire data in both longitudinal and transverse orientations with respect to the AoS, which was indicated by the muscle fibers’ long axes. VisR data were acquired using a Siemens S3000 Helix imaging system with a 9-L4 transducer (Siemens Healthineers, Ultrasound Division, Issaquah, WA, USA). The transducer was mounted on a 6-DoF serial robotic arm (Meca500, Mecademic, Montreal, Quebec, Canada), which was used to acquire data starting at normal (90°) ARF-AoS incidence, followed by 10°, 20°, 30°, and 40° tilting of the transducer about a fixed center axis (corresponding to 80°, 70°, 60° and 50° ARF-AoS incidence angles) in both longitudinal and transverse orientations. This acquisition configuration is illustrated in Fig. 3. Three separate VisR acquisitions were repeated at each location without moving the transducer.

For each scan, the imaging focal depths in the chicken and bovine samples were 20 mm and 30 mm, respectively, due to the thickness of the specific samples. Apart from the focal depths, the same imaging parameters (center frequencies, focal configurations, ARF pulse duration, PRF) outlined in Table 1 for *in silico* data generation were implemented for the *ex vivo* data acquisitions. VisR data were collected in ensembles consisting of two reference pulses, two ARF impulses with 6 tracking lines between, and then additional tracking lines. The ensembles of VisR data were acquired in 40 lateral positions evenly spaced across a 2 cm lateral field-of-view (FOV) for 2D imaging. ARF-induced displacements were measured using 1D NCC, also with the same parameters listed in Table 1 as with the *in silico* experiments. The temporal displacement profiles were then fit to the MSD model using non-linear least squares minimization, as described above, to form 2D parametric images of VisR RE for each acquisition. From these parametric images, VisR RE was analyzed in terms of mean and standard deviation in a 2 mm ROI about the focal depth across the 3 acquisitions taken at each location.

## III. RESULTS

**FIGURE 4.**
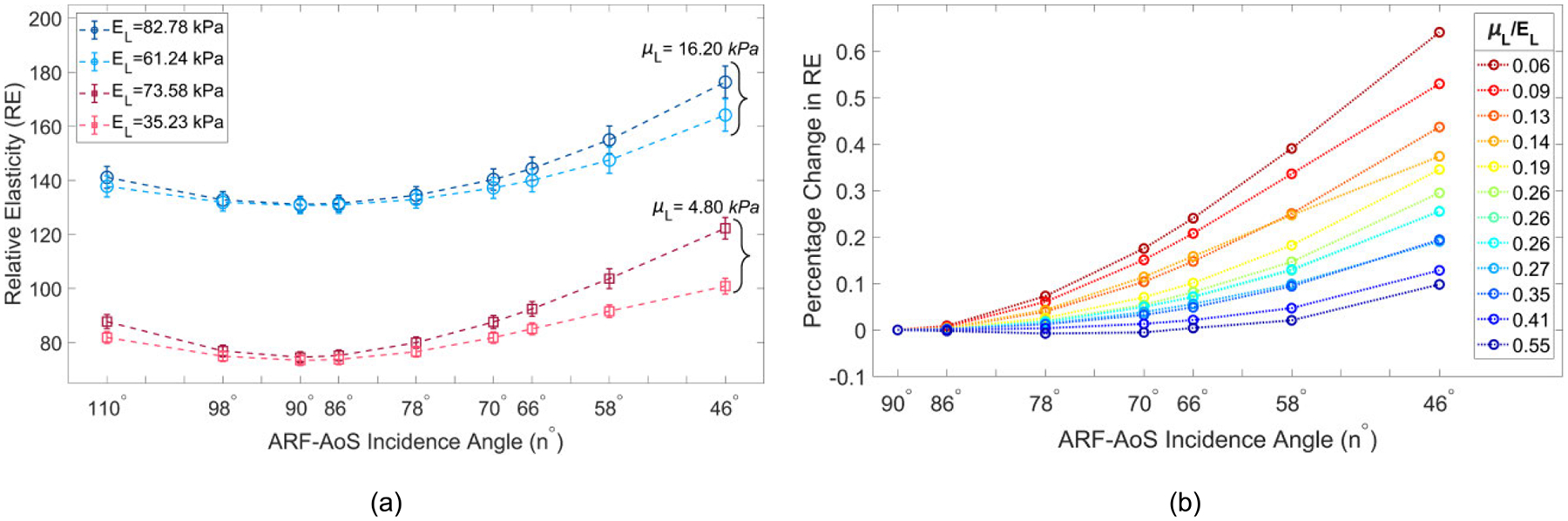
(a) RE versus ARF-AoS incidence angle in simulated TI materials clustered by the same $\mu_{L}$ with the transducer aligned to interrogate the plane of symmetry (longitudinal orientation); (b) percent change in RE (ΔRE) compared to the RE measured at 90° versus ARF-AoS incidence angle.

Fig. 4(a) illustrates VisR RE versus ARF-AoS incidence angle for longitudinal orientation in four simulated TI materials. The materials are clustered by longitudinal shear modulus values, with two materials (blue shades) having $\mu_{L}=16.20\;\text{kPa}$ and two materials (red shades) having $\mu_{L}=4.80\;\text{kPa}$. Three observations are notable. First, when the ARF-AoS incidence angle is 90°, RE varies by shear elastic modulus only. Second, as the ARF-AoS incidence angle deviates (increases or decreases) from 90°, RE increases. Finally, for a given shear elastic modulus, the degree of this RE increase is greater in the material with the larger Young’s modulus.

For all simulated materials, the percentage change in RE at different ARF- AoS incidence angles compared to the RE measured at 90° was calculated and defined as ΔRE. Fig. 4(b) shows ΔRE versus ARF-AoS incidence angle for materials with different ratios of longitudinal shear-to-Young’s elastic moduli $\left(\mu_{L}/E_{L}\right)$. Materials with lower $\mu_{L}/E_{L}$ exhibited greater percent change in RE for a given change in ARF-AoS incidence angle. The statistical relationship between ΔRE and $\mu_{L}/E_{L}$ was assessed using Spearman’s correlation test. The correlation coefficients, reported in Table 2, indicate a strong correlation, particularly for 78° and smaller incidence angles.

**FIGURE 5.**
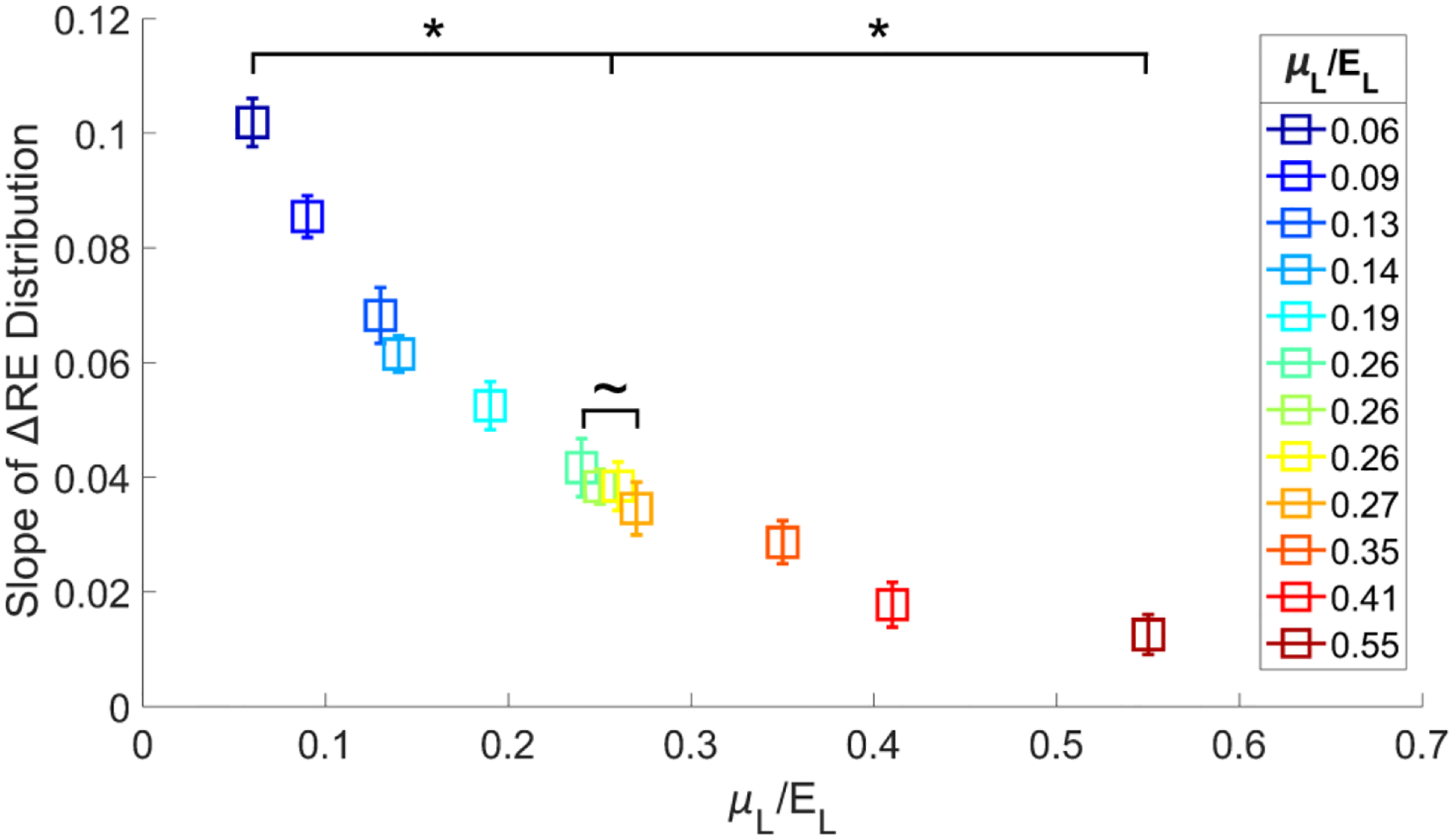
Slopes of ΔRE versus ARF-AoS incidence angle for each material from Fig. 4(b), plotted against the corresponding materials’ $\mu_{L}/E_{L}$ ratios. Statistical difference between the slopes of materials with adjacent $\mu_{L}/E_{L}$ values, as assessed by Wilcoxon rank sum tests (with 95% confidence interval), is indicated by ‘*’.

To quantify the relationship between ΔRE and $\mu_{L}/E_{L}$, linear regression was performed on the ΔRE versus ARF-AoS incidence angle data for each material from Fig. 4(b). The slopes of the fitted lines, representing the rate of change of ΔRE with respect to ARF-AoS incidence angle, were then extracted. These slopes were plotted against the corresponding $\mu_{L}/E_{L}$ ratios of the materials, as shown in Fig. 5. It can be observed that slope decreases with increasing $\mu_{L}/E_{L}$ ratio. Wilcoxon rank sum tests (with 95% confidence intervals) indicated that the slopes of materials with the same or similar $\mu_{L}/E_{L}$ values (0.26, 0.26, 0.26, and 0.27) were not statistically different, while the slopes of all other materials with adjacent $\mu_{L}/E_{L}$ values were statistically different.

**FIGURE 6.**
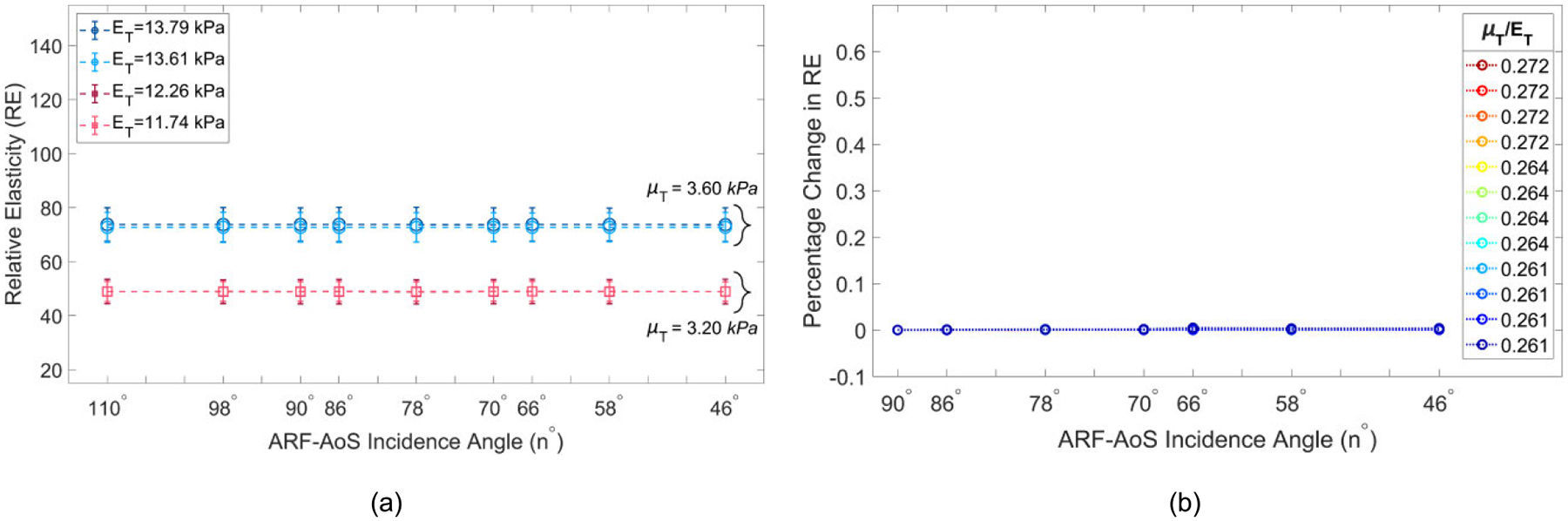
(a) RE versus ARF-AoS incidence angle in simulated TI materials clustered by the same $\mu_{T}$ with the transducer aligned to interrogate the plane of isotropy (transverse orientation); (b) percent change in RE (ΔRE) compared to the RE measured at 90° versus ARF-AoS incidence angle.

Fig. 6(a) illustrates VisR RE versus ARF-AoS incidence angle for transverse orientation in 4 simulated TI materials. The materials are clustered by transverse shear modulus values, with two materials (blue shades) having $\mu_{T}=3.60\;\text{kPa}$ and two materials (red shades) having $\mu_{T}=3.20\;\text{kPa}$. It can be observed that RE varies by $\mu_{T}$ but is not impacted by the ARF-AoS incidence angle. Furthermore, from Fig. 6(b) it is notable that ΔRE values are negligible across all simulated materials regardless of the ARF-AoS incidence angle.

**FIGURE 7.**
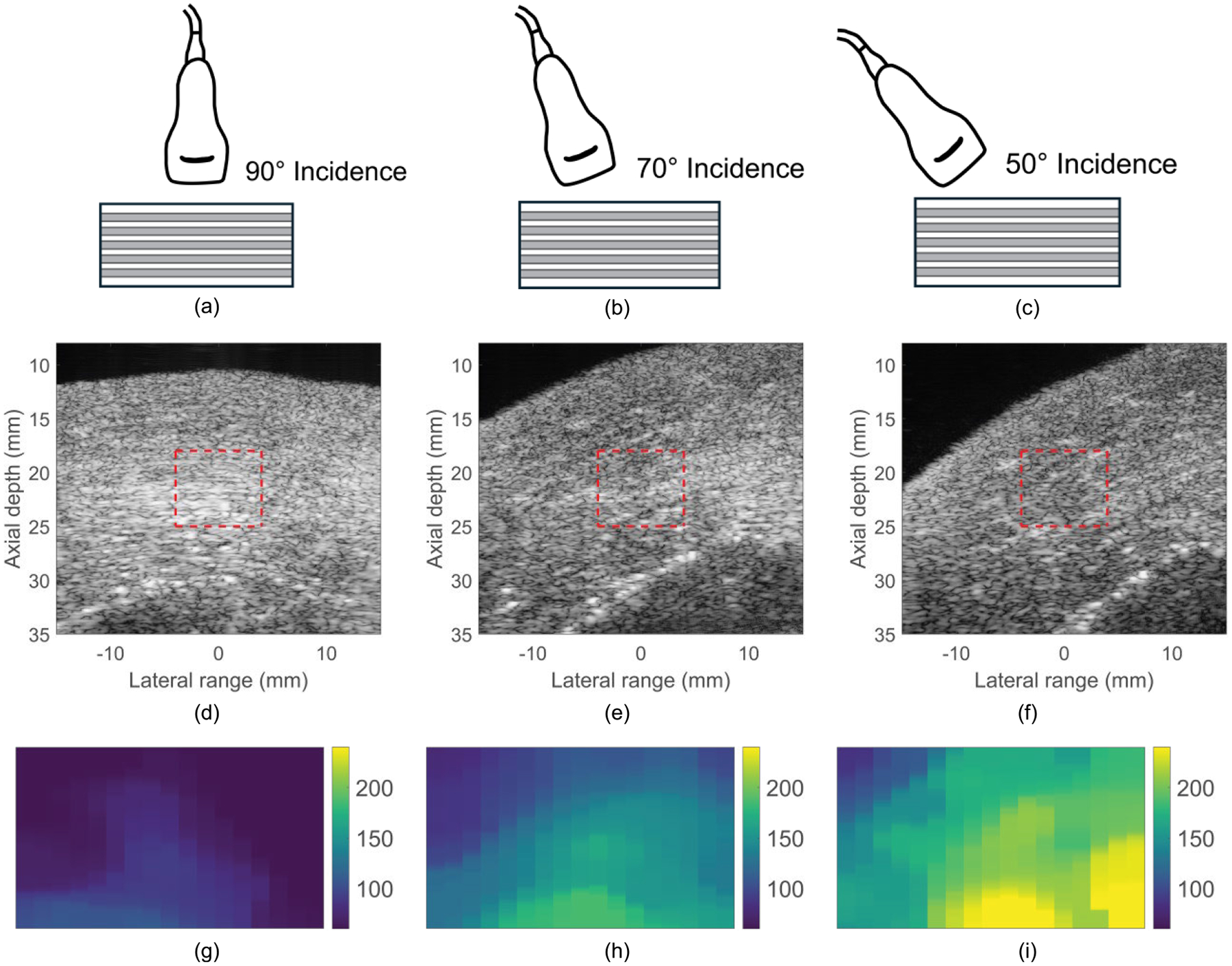
Transducer positioning for ARF-AoS incidence angles of (a) 90°, (b) 70°, and (c) 50° degrees in longitudinal orientation in a TI material; B-mode images of chicken *pectoralis major* at ARF-AoS incidence angles of (d) 90°, (e) 70°, and (f) 50° in longitudinal orientation; and parametric RE images of chicken *pectoralis major* at ARF-AoS incidence angles (g) 90°, (h) 70°, and (i) 50° in longitudinal orientation. These RE images were generated from the regions marked by red rectangles in the corresponding B-mode images.

Fig. 7 illustrates B-mode and parametric RE images of chicken *pectoralis major* at ARF-AoS incidence angles of 90°, 70°, and 50° in longitudinal orientation. Across the incidence angles, variation in the muscle fiber alignment with respect to the transducer can be seen in the B-mode images. The corresponding parametric RE images demonstrate a progressive increase in measured elasticity as the incidence angle decreases from 90°.

**FIGURE 8.**
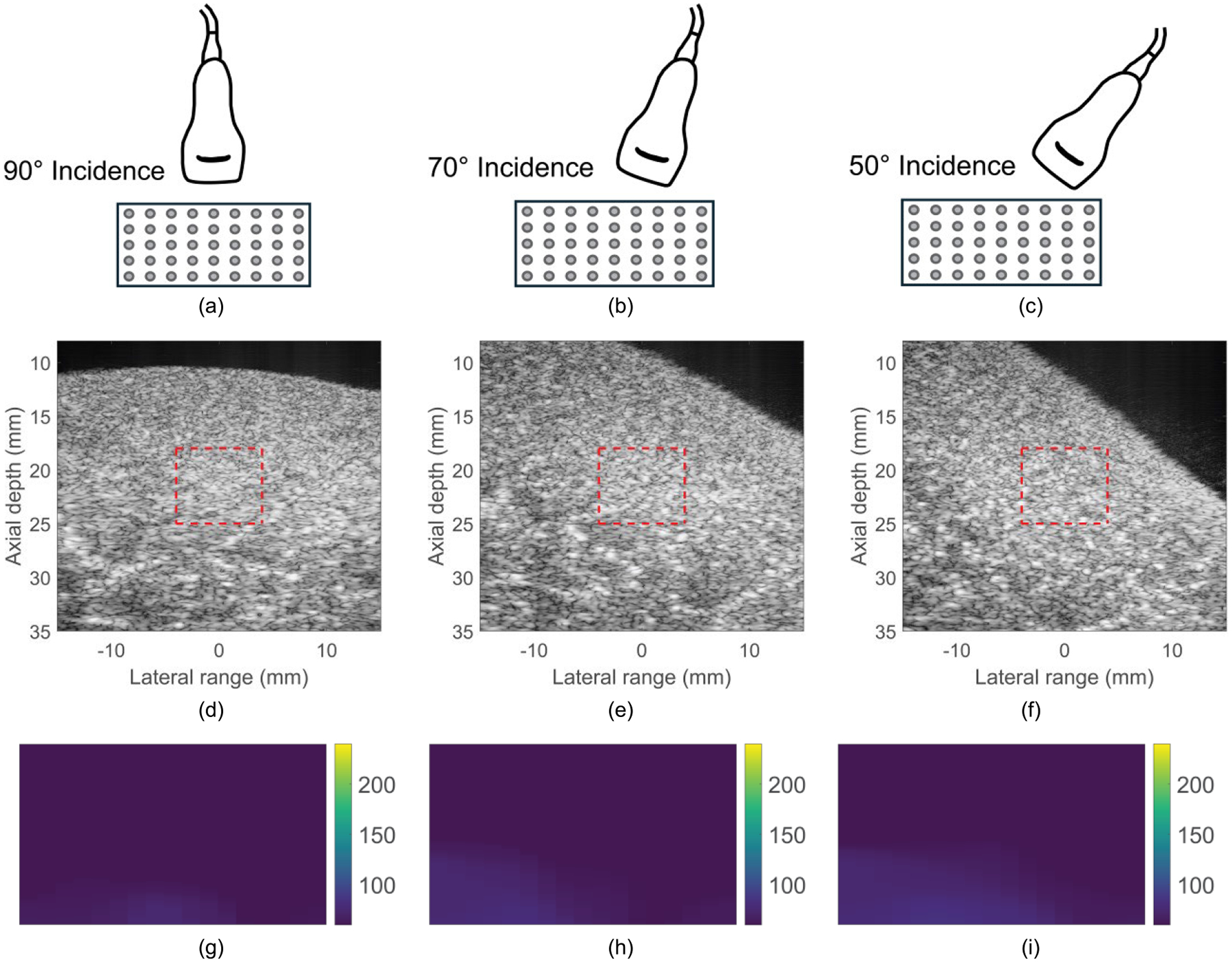
(a) Transducer positioning for ARF-AoS incidence angles of (a) 90°, (b) 70°, and (c) 50° degrees in transverse orientation in a TI material; B-mode images of chicken *pectoralis major* at ARF-AoS incidence angles of (d) 90°, (e) 70°, and (f) 50° in transverse orientation; and parametric RE images of chicken *pectoralis major* at ARF-AoS incidence angles (g) 90°, (h) 70°, and (i) 50° in transverse orientation. These RE images were generated from the regions marked by red rectangles in the corresponding B-mode images.

Fig. 8 demonstrates B-mode and parametric RE images obtained in transverse orientation for the same chicken sample. It can be observed that the measured elasticity is nearly constant across the different incidence angles.

**FIGURE 9.**
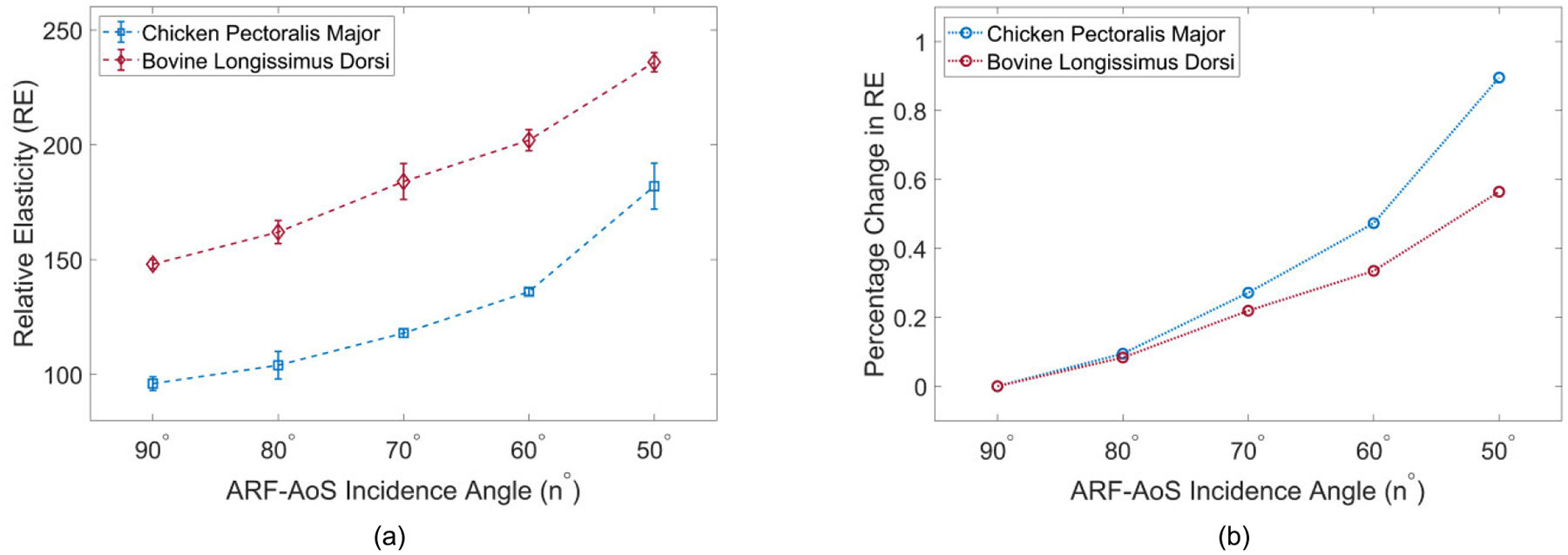
(a) VisR RE versus ARF-AoS incidence angle in *ex vivo* chicken *pectoralis major* and bovine *longissimus dorsi* samples, with the transducer oriented to interrogate the plane of symmetry (longitudinal orientation), and (b) corresponding percent change in RE versus ARF-AoS incidence angle (ΔRE) for both samples.

**FIGURE 10.**
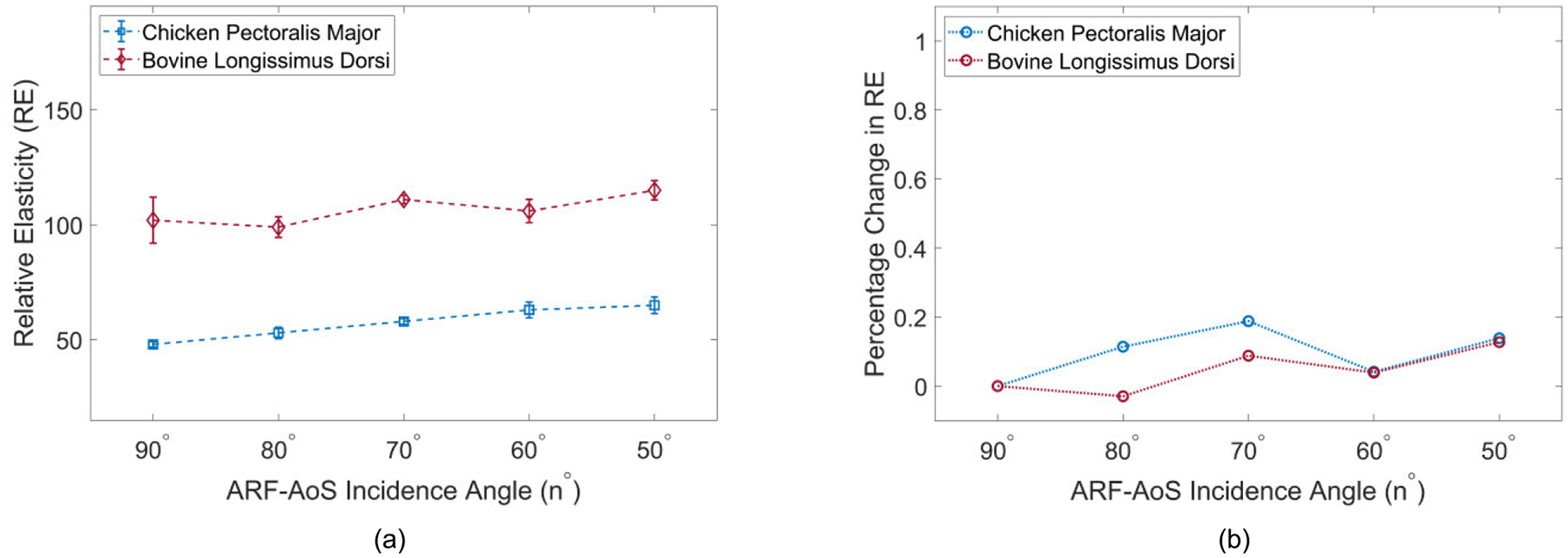
(a) VisR RE versus ARF-AoS incidence angle in *ex-vivo* chicken *pectoralis major* and bovine *longissimus dorsi* samples, with the transducer oriented to interrogate the plane of isotropy (transverse orientation), and (b) corresponding percent change in RE versus ARF-AoS incidence angle (ΔRE) for both samples.

Fig. 9(a) illustrates VisR RE versus ARF-AoS incidence angle for longitudinal orientation in both chicken *pectoralis major* and bovine *longissimus dorsi*. Percent change in RE relative to RE at 90° ARF-AoS incidence for both tissues is illustrated in Fig. 9(b). It is notable that ΔRE increases more rapidly for chicken *pectoralis major* than for bovine *longissimus dorsi*. Figure 10 similarly shows VisR RE (panel (a)) and $\Delta RE\left(n^{\circ }\right)$ (panel (b)) versus ARF-AoS incidence angle for both chicken and bovine samples, but for transverse orientation interrogating the plane of isotropy. Consistent with *in silico* results, VisR RE was nearly constant as the ARF-AoS incidence angle varied.

**FIGURE 11.**
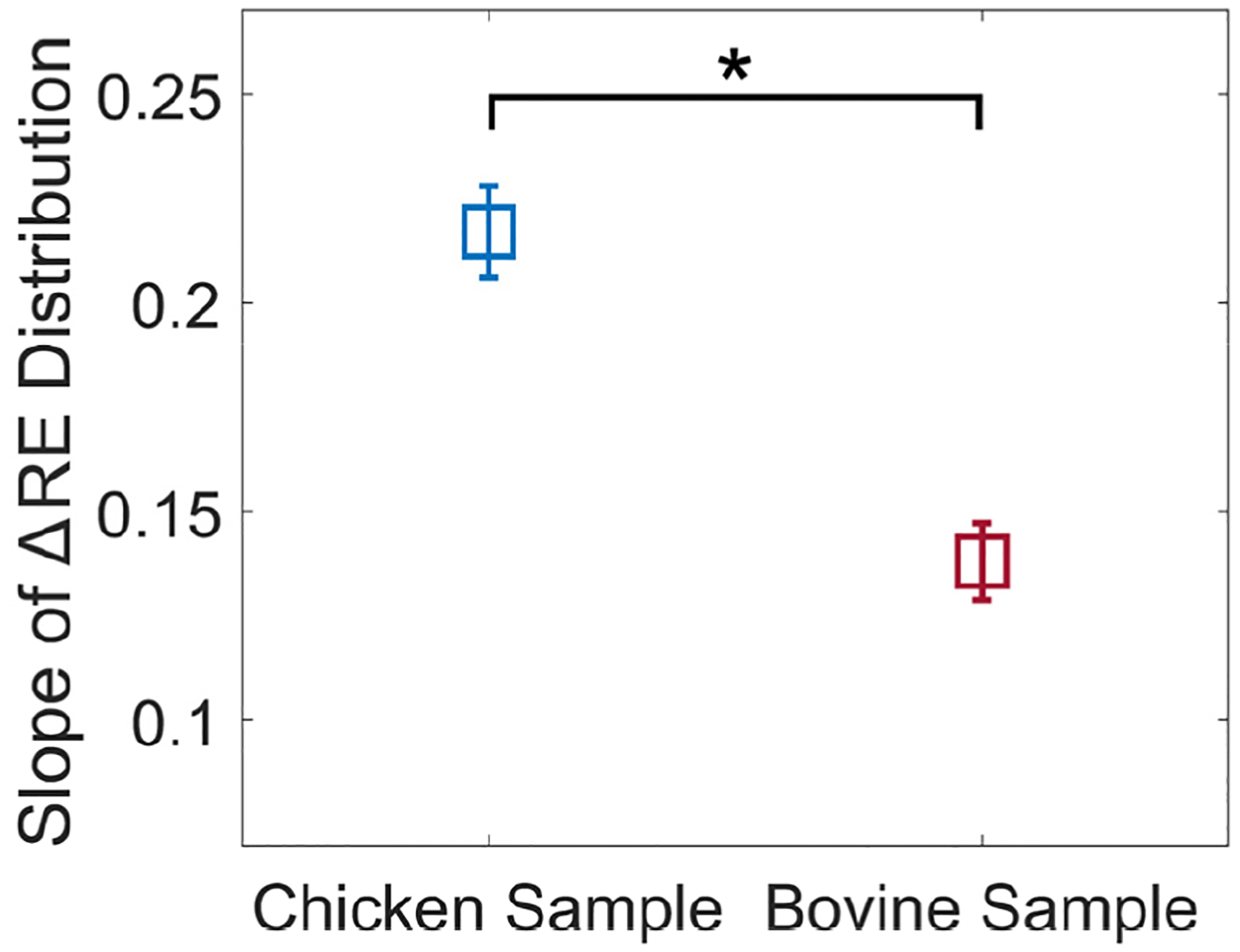
Slopes of linear regression lines fit to ΔRE versus ARF-AoS incidence angle for chicken *pectoralis major* and bovine *longissimus dorsi* in longitudinal orientation. Statistical difference (Wilcoxon rank sum tests with 95% confidence interval) indicated by ‘*’.

Fig. 11 illustrates the slopes of linear regression lines fit to ΔRE versus ARF-AoS incidence angle for both chicken and bovine samples in longitudinal orientation (Fig. 9(b)). Wilcoxon rank sum tests (95% confidence interval) indicated that the slopes for chicken and bovine muscles were statistically significantly different (p<0.05, indicated by ‘*’).

## IV. DISCUSSION

This study evaluated the potential for VisR ultrasound to interrogate both shear and Young’s elastic moduli in TI materials using non-normal ARF-AoS incidence angles. Figures 4, 7, and 9 demonstrate that VisR RE measurements taken using non-normal ARF-AoS incidence angles in the plane of symmetry vary with ARF-AoS incidence angle to a degree that is proportional to the longitudinal Young’s modulus $\left(E_{L}\right)$. On the contrary, VisR measurements taken in the plane of isotropy do not vary with ARF-AoS incidence. These results have several meaningful implications.

First, the result that percent change in RE versus ARF-AoS incidence angle in the plane of symmetry is highly correlated to the underlying ratio of $\mu_{L}/E_{L}$ (Fig. 4(b) and Table 2) highlights the opportunity to interrogate $\mu_{L}/E_{L}$ as an independent biomarker. The potential relevance of such is demonstrated in Fig. 9(b), in which chicken and bovine muscles were differentiated by trends in RE. Considering the data in Fig. 9 more carefully, bovine muscle had higher RE at 90° ARF-AoS incidence in both the plane of symmetry (suggesting a higher $\mu_{L}$) and in the plane of isotropy (Fig. 10(a), suggesting a higher $\mu_{T}$), and the percent change in RE with ARF-AoS incidence in the plane of symmetry was lower (suggesting a larger $\mu_{L}/E_{L}$) relative to chicken. The relevance of interrogating $\mu_{L}/E_{L}$ is further demonstrated in Figs. 5 and 11, in which the linear regression slope of change in RE versus ARF-AoS incidence angle, a semi-quantitative parameter, statistically discriminated TI materials with different $\mu_{L}/E_{L}$ *in silico* and chicken and bovine muscle *ex vivo*. These results demonstrate that obtaining VisR elasticity measurements across a range of ARF-AoS incidence angles in the plane of symmetry can enable interrogation of $\mu_{L}/E_{L}$ in anisotropic tissues. The clinical relevance of this finding will be evaluated in future studies.

Second, variations in the ARF-AoS incidence were herein achieved using a combination of beam tilting and material tilting *in silico* and transducer tilting *ex vivo*. While electronic beam steering would enable more efficient data collection, the steering range of ±20° that is typical for commercial systems would not support the wide range of ARF-AoS incidence angles used in this study. The sufficiency of an ARF-AoS incidence range of ±20° for interrogating $\mu_{L}/E_{L}$ would need to be determined. Although Table 2 denotes that ΔRE is highly correlated with $\mu_{L}/E_{L}$ at incidence angles as close to 90° as 78° (12° steering), from Fig. 4(b), ΔRE distinguishes between materials with $\mu_{L}/E_{L}$ that are close in value when the ARF-AoS incidence angle is 58° or smaller (32° steering or more). Consistent results were obtained *ex vivo* (Fig. 9), when ΔRE was able to differentiate between chicken and bovine tissues at 60° and 50° ARF-AoS incidence angles, suggesting that about ±30° steering may be needed. An additional complication introduced by electronic steering is the associated alterations to beam properties [34], including changes in beam shape, focal position, prominence of side lobes, etc. The impact of such changes on VisR RE measurements will be evaluated in future work. If it is ultimately determined that electronic steering is not optimal, then specialized devices to support efficient mechanical steering could be designed to make *in vivo* data collection by mechanical steering convenient.

Third, VisR ultrasound derived RE provides an inherently qualitative indication of elasticity. This is because RE values are relative to the applied ARF amplitude, which is assumed to remain constant but is inherently unknown. As such, absolute RE values cannot be directly compared across different specimens or patients unless the applied ARF amplitude is verified to be constant across acquisitions. To address this limitation, this study focuses on the percentage change in RE (ΔRE) as a function of ARF-AoS incidence angle within the same specimen. Furthermore, we parameterize the ΔRE distributions by fitting a linear regression model to these distributions and evaluating the slope. This slope represents the rate of change in ΔRE across incidence angles and serves as a semi-quantitative parameter (not fully quantitative—being proportional to $\mu_{L}/E_{L}$ rather than equal to $\mu_{L}/E_{L}$), providing a consistent metric for comparisons across specimens or patients. However, employing quantitative on-axis elastography techniques, such as Double Profile Intersection (DoPIo) [35], [36] or Quantitative VisR (QVisR) [37], may enable relating the absolute differences in modulus estimates across a range of ARF-AoS incidence angles directly to the underlying $E_{L}$. This can potentially lead to quantification of both $\mu_{L}$ and $E_{L}$ in the plane of symmetry, and $\mu_{T}$ in the plane of isotropy for more complete characterization of anisotropic tissues.

An important additional outcome of this work is the knowledge that VisR assessments of longitudinal shear elastic modulus $\left(\mu_{L}\right)$, which are made in longitudinal orientation, could be confounded by the longitudinal Young’s modulus $\left(E_{L}\right)$ if the radiation force is not delivered normal to the underlying AoS. This is especially important to consider when evaluating anisotropic tissues *in vivo* because the underlying AoS may not be aligned with the skin surface, such as in bipennate muscles [38], [39] or in the context of altered collagen organization caused by cancers [40], [41] or other pathologies [42]. However, the results in Fig. 4 also suggest that, in such cases, it would be possible to electronically or mechanically sweep data acquisitions to identify the angle that provides the lowest elasticity measure, as our findings demonstrate that normal radiation force incidence is associated with the minimum value of RE (Figs. 4, 7, and 9). Alternatively, the confounding effects of non-normal ARF-AoS incidence can be avoided by performing VisR in only the plane of isotropy with transverse orientation, as shown in Figs. 6, 8, and 10, but this would limit elasticity characterization to only the transverse shear elastic modulus $\left(\mu_{T}\right)$.

A limitation in the study design was that *in silico* results were obtained from a collection of 12 simulated TI materials. Although the elasticities of the evaluated materials spanned the wide range of values expected for various types of soft tissue, including skeletal muscle, kidney and breast, a larger investigation using more materials could help to clarify optimal ARF-AoS incidence angle range and step-size, as well as the potential for electronic steering in clinical settings. Another limitation was that tissue experiments were conducted *ex vivo* with no aberrating layer between the transducer and the target tissue. Defocusing from aberration (or other causes) would widen the ARF excitation, resulting in a stronger contribution from $\mu_{T}$ in VisR RE measures in the plane of symmetry, which could decrease sensitivity to $\mu_{L}/E_{L}$. Future studies will include aberrating layers and consider the impact of increasing the ARF focal configuration. Additionally, ΔRE distributions were parameterized using a linear regression model, which may not always perfectly capture the underlying relationship between the percentage change in RE across ARF-AoS incidence angles. While this simple approach was effective in distinguishing TI materials in this study, future work will explore more complex models that could better represent the observed data and further refine the semi-quantitative parameterization.

## V. CONCLUSION

This study has shown that VisR ultrasound can be used to interrogate both shear and Young’s elastic moduli in TI materials. *In silico* findings within the plane of symmetry revealed that VisR RE measurements taken at non-normal ARF-AoS incidence angles were impacted by both the longitudinal Young’s $\left(E_{L}\right)$ and shear ${(\mu }_{L})$ elastic moduli. Moreover, the percent change in RE with varying incidence angles (ΔRE) was proportional to the $\mu_{L}/E_{L}$ ratio. Furthermore, the slopes of linear regression lines fit to the percent change in RE versus ARF-AoS incidence angle distributions demonstrated a strong correlation with the underlying $\mu_{L}/E_{L}$ of the materials, and these slopes were able to statistically differentiate between TI materials with varying $\mu_{L}/E_{L}$ ratios. It was found that in the plane of isotropy, VisR elasticity measurements remained constant over ARF-AoS incidence angle. These *in silico* results were translated to *ex vivo* application in chicken *pectoralis major* and bovine *longissimus dorsi* muscles. The rate of change of ΔRE with incidence angle, indicative of $\mu_{L}/E_{L}$, distinguished between the two tissues with statistical significance. These findings suggest that the rate of change of ΔRE with incidence angle has the potential to serve as a novel semi-quantitative biomarker for evaluating anisotropic tissues such as kidney, skeletal muscle, and breast.

## Figures and Tables

**TABLE 1.** Parameters used to simulate the ARF field and ultrasonic tracking, as well as NCC parameters used for displacement tracking. The same parameters were also used for experimental acquisitions.

	Parameter Name	Value
**Imaging**	Sampling frequency	40 MHz
	Focal depth	20 mm
**ARI excitation**	Duration	300 cycles
	Center frequency	4.21 MHz
	Focal configuration	1.5
**Tracking**	Center frequency	6.15 MHz
	Transmit focal configuration	1.5
	Receive focal configuration	0.75*
	Pulse Repetition Frequency	10 KHz
**Normalized Cross Correlation**	Kernel length	512 μm
	Search region	80 μm

* Aperture growth and dynamic Rx focusing enabled

**TABLE 2.** Spearman correlation coefficients between ΔRE and $\mu_{L}/E_{L}$ at each incidence angle in the plane of symmetry.

ΔRE	Correlation with $\mu_{L}/E_{L}$
ΔRE(86°)	−0.63
ΔRE(78°)	−0.98
ΔRE(70°)	−0.97
ΔRE(66°)	−0.97
ΔRE(58°)	−0.98
ΔRE(46°)	−0.99
